# Mesoscopic Fluorescence Imaging of Light-Triggered Chemotherapeutic Release in Cancer Spheroid Models

**DOI:** 10.3390/pharmaceutics18040495

**Published:** 2026-04-17

**Authors:** Elias Kluiszo, Rasel Ahmmed, Berna Aliu, Semra Aygun-Sunar, Matthew Willadsen, Hilliard L. Kutscher, Jonathan F. Lovell, Ulas Sunar

**Affiliations:** 1 Department of Biomedical, Engineering, Stony Brook University, Stony Brook, NY 11794, USA; elias.kluiszo@stonybrook.edu (E.K.); rasel.ahmmed@stonybrook.edu (R.A.); berna.aliu@stonybrook.edu (B.A.); semra.aygun@stonybrook.edu (S.A.-S.); 2 POP Biotechnologies, Buffalo, NY 14228, USA; mwilladsen@popbiotech.com (M.W.);; 3 Department of Biomedical Engineering, University at Buffalo, Buffalo, NY 14260, USA; jflovell@buffalo.edu; 4 Department of Electrical and Computer Engineering, Stony Brook University, Stony Brook, NY 11794, USA

**Keywords:** chemophototherapy, light-triggered drug release, porphyrin-phospholipid liposomes, doxorubicin, fluorescence-guided drug delivery, theranostics, tumor spheroid model, peritoneal micrometastases, laparoscopic fluorescence imaging, quantitative fluorescence imaging, head and neck cancer, oral squamous cell carcinoma

## Abstract

**Background/Objectives:** Peritoneal micrometastases (micromets) remain a major barrier to durable cytoreduction in ovarian and other intra-abdominal cancers because lesions are difficult to visualize and are often resistant to systemic therapy. Liposomal doxorubicin (Dox) improves pharmacokinetics but can be limited by slow intratumoral release. Porphyrin-phospholipid (PoP) liposomes enable near-infrared light–triggered release of Dox (chemophototherapy (CPT)), creating an opportunity for intraoperative fluorescence-guided treatment planning and monitoring. Here, we evaluate a laparoscopic fluorescence imaging platform for quantifying light-triggered drug delivery. **Methods:** LC-Dox-PoP was applied to SCC2095sc and SKOV-3 cultures in 2D monolayers and 3D spheroid clusters. Dox fluorescence was quantified using a laparoscopic fluorescence imaging system over 1–9 μg/mL concentrations and compared with standard well-plate reader measurements. Porphyrin fluorescence was monitored to assess spheroid localization and photobleaching after activation light exposure. **Results:** For both cell lines, Dox fluorescence exhibited an approximate 4-fold increase at the maximum administered LC-Dox-PoP concentration, following a linear trend in both SCC2095sc and SKOV-3 cultures (R^2^ = 0.97, 0.98 for 2D and R^2^ = 0.98, 0.98 for spheroids). Laparoscope-derived fluorescence measurements agreed with well-plate reader measurements (R^2^ = 0.89–0.96). Porphyrin fluorescence provided stronger complementary contrast for localizing spheroid constructs and decreased after activation light exposure, consistent with photobleaching during triggered release. **Conclusions:** These results support a quantitative imaging framework for fluorescence-guided monitoring of light-triggered liposomal drug release and may enable individualized CPT dosimetry for peritoneal micrometastases. Findings in SCC2095sc additionally suggest potential relevance of fluorescence-guided CPT for head and neck/oral cancer, where localized post-resection adjuvant treatment may improve control of residual disease.

## 1. Introduction

Peritoneal micrometastases (micromets) represent significant hurdles in the successful treatment of intra-abdominal malignancies, specifically ovarian cancer, which remains one of the most lethal gynecological diseases due to high recurrence rates [1,2,3]. These micromets are often too small to be detected by conventional imaging techniques such as computed tomography (CT), magnetic resonance imaging (MRI), positron emission tomography (PET), and ultrasound, which often demonstrate lower sensitivity than reassessment surgeries [4,5,6,7]. Consequently, even after surgical resection and adjuvant chemotherapy, up to 60% of patients may still harbor occult disseminated disease [1,2,4,6,8]. Thus, there is an urgent need for sensitive intraoperative imaging methods that allow surgeons to visualize and selectively treat these lesions in real-time while avoiding damage to healthy tissue.

An emerging solution lies in chemophototherapy (CPT), which combines light-activated drug release with photodynamic effects [9,10,11,12,13,14]. While traditional systemic chemotherapy often leads to significant toxic side effects [12,14,15,16,17,18,19], these liposomal nanocarriers improve the biodistribution and efficacy of the payload [19,20]. In our approach, doxorubicin (Dox) is encapsulated within porphyrin-phospholipid (PoP) liposomes, improving biodistribution while enabling spatially confined, near-infrared light–triggered release (~660 nm) at the illuminated site [9,10,15,16]. The lipid envelope protects the drug from degradation and facilitates passive accumulation at tumor sites via the enhanced permeability and retention (EPR) effect [5,21,22]. In parallel, photodynamic therapy (PDT) leverages light, a photosensitizing agent, and molecular oxygen to generate cytotoxic reactive oxygen species that can selectively damage tumor tissue [21,23]. Porphyrin-based photosensitizers preferentially accumulate in malignant cells, making PDT itself a promising surgical adjunct for treating ovarian micromets [14,24,25,26,27,28]. They have been used in many human clinical trials and naturally accumulate 2–3 fold higher in malignant cells [16,17]. Targeting strategies (e.g., EGFR- or folate-directed) have been shown to reduce off-target peritoneal phototoxicity in preclinical models [2,6,14,23,29,30,31,32,33,34]. Together, these features provide a controlled-release, site-specific drug delivery platform that may improve micromet treatment while minimizing systemic toxicity. Optically triggered combination regimens that integrate imaging and PDT have been positioned as intrinsically theranostic approaches for treatment planning and monitoring [35]. Related imaging-guided phototherapies have leveraged theranostic nanoliposomal photosensitizer formulations and dual-function photosensitizer–fluorophore constructs for fluorescence-guided PDT, supporting the broader “see-and-treat” paradigm for treatment planning and monitoring [36,37].

A primary advantage of this approach is the fluorescence properties of both the PoP carrier and the doxorubicin payload, enabling high-sensitivity, image-guided delivery. PoP fluorescence (~720 nm) can localize nanocarrier accumulation, supporting lesion targeting and treatment planning, while Dox fluorescence (~590 nm) can serve as a readout of drug kinetics and light-triggered release at the target [15,16]. By leveraging a laparoscope equipped with appropriate excitation and emission filtering, these markers can support real-time visualization and quantitative monitoring during minimally invasive CPT.

However, accurately quantifying drug fluorescence concentrations in vivo remains challenging due to heterogeneous tissue optical properties and depth-dependent attenuation. Quantitative approaches such as wide-field and laparoscopic/endoscopic spatial frequency domain imaging (SFDI) can map optical properties and support fluorescence correction in scattering tissue [38,39,40,41,42]. To bridge the gap between monolayer cell cultures and complex animal models, 3D multicellular spheroid systems provide a critical intermediate platform. Unlike traditional 2D models, spheroids replicate key transport barriers and cellular architecture relevant to peritoneal micrometastases, enabling controlled evaluation of drug distribution, penetration, and fluorescence readouts in a geometry that mimics occult micromet clusters [43,44,45].

In this work, we present a mesoscopic evaluation of light-triggered drug delivery using a custom-designed laparoscopic fluorescence imaging system. This platform was used to monitor uptake and 660 nm-triggered release of long-circulating Dox-PoP (LC-Dox-PoP) liposomes in 3D multicellular spheroid cluster models. By imaging across multiple administered concentrations, we calibrated doxorubicin-associated fluorescence and characterized uptake and release dynamics in spheroids, capturing the transition from the encapsulated state to the therapeutically active diffusive state. Together, these studies establish mesoscopic laparoscopic imaging as a quantitative framework for treatment planning and monitoring of CPT in peritoneal micrometastases. To assess generalizability beyond ovarian cancer, we also evaluate SCC2095sc spheroid clusters, an oral squamous cell carcinoma model relevant to head and neck/oral cancer. Recent head and neck/oral squamous cell carcinoma studies have used 3D spheroid models for quantitative evaluation of image-guided phototherapies, supporting the translational relevance of spheroid platforms for CPT optimization [46,47,48].

## 2. Materials and Methods

### 2.1. Laparoscopic SFDI System

**Figure 1:**
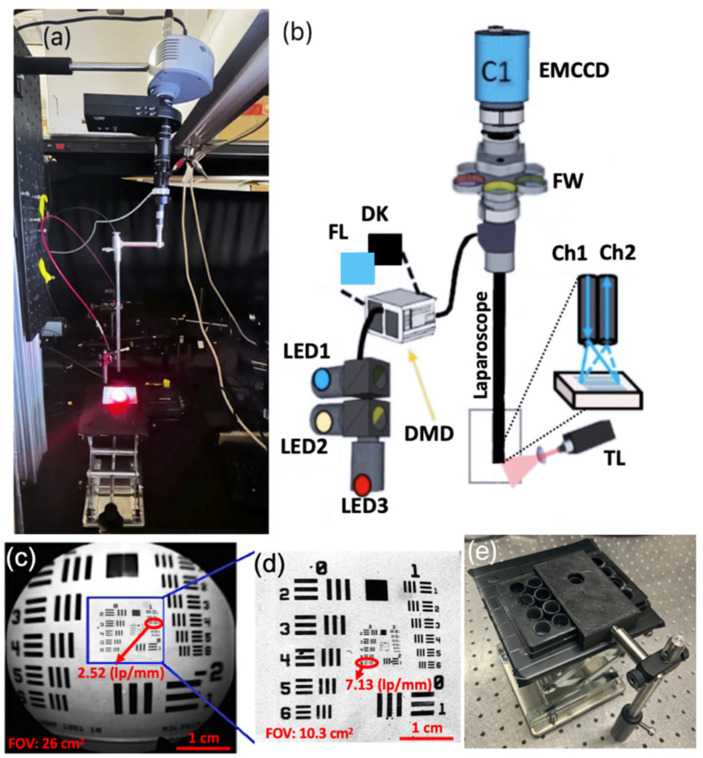
The experimental setup of doxorubicin release in a cell spheroid model: (**a**) laparoscope SFDI system picture projecting excitation light and the 660 nm treatment light in red, (**b**) laparoscope SFDI setup design sketch, showing component details, and (**c**) laparoscopic fluorescence imaging of a United States Air Force (USAF) target. (**d**) Reflectance imaging of the same target. The blue box in (**c**) demonstrates the relative scale used for both the fluorescence and reflectance modalities. (**e**) Custom well-plate holder with a fixed viewing port and sliding track to ensure consistent spatial alignment of the well plate across sequential imaging sessions.

The laparoscopic SFDI system, previously validated in [49], is shown in Figure 1. Specifically, Figure 1a depicts the sample imaging arrangement for the release experiment. Meanwhile, Figure 1b highlights the internal laparoscopic components, including the imaging elements and the digital micromirror device (DMD) used for projection.

To illuminate the laparoscopic fluorescence imaging system, three high-power LEDs with wavelengths of 490 nm, 590 nm, and 656 nm were utilized. These were coupled to the DMD via a liquid light guide (Mightex, ON, Canada). The operation of the LED and DMD components was remotely controlled using a customized MATLAB 2024a (Natick, MA, USA) interface. Excitation light was projected through a 2.4 mm imaging fiber containing 13,000 elements (Asahi Kasei, Tokyo, Japan), which was coupled to a fixed laparoscope and delivered light to the distal end facing the tissue. A custom objective lens at the fiber tip collimated the output and provided uniform illumination across the tissue surface. The excitation signals were collected through the laparoscope optics, including the R. Wolf laparoscope, model 8912.43 (Vernon Hills, IL, USA), a zoom coupler (Accu-Beam, TTI Medical, San Ramon, CA, USA), two 25 mm achromatic lenses, a filter wheel, and an aperture (ThorLabs, Newton, NJ, USA), before being detected by an EMCCD camera (1004 × 1002 pixels, Luca, Andor, Belfast, Ireland). With a 5.1 × 5.1 cm field of view (FOV), the system successfully resolved group 1, element 3 of the target, which represents a line width of 198.43 μm (2.52 lp/mm). This performance, illustrated in Figure 1c, aligns with the typical 2–5 lp/mm resolution range [50] of standard high-definition laparoscopes (Table 1). At a higher magnification shown in Figure 1d (using a smaller 3.2 × 3.2 cm FOV), the laparoscopic system achieved a resolution of group 2, element 6, corresponding to a line width of 70.15 μm or 7.13 lp/mm.

A 490 nm LED with a 490 ± 10 nm bandpass filter was used to excite Dox fluorescence, and the 656 nm LED was used to excite porphyrin fluorescence. To isolate the fluorescent signal of doxorubicin, a 530 nm long-pass filter and a 593 ± 40 nm fluorescent filter were used. Neutral density (ND) filters, matched to the transmission of the fluorescent filters, are used to obtain reflectance data and remove specular reflections and signal leakages, as described further in 2.4. To capture fluorescent signal from the porphyrin-loaded liposomes, a 750 ± 50 nm fluorescent filter was used with the 656 nm excitation light. A custom 3D-printed black structure (Figure 1e) serves as a physical stop for multi-well plates. This ensures precise alignment and a constant imaged region across successive captures and filter changes while mitigating spectral reflections.

### 2.2. Preparation of Long-Circulating Dox in PoP Liposomes

The specifics of our doxorubicin-loaded PoP-liposome formulation are based on methods previously detailed in [1,4,38]. Demonstrating the platform’s versatility, PoP-liposomes were recently utilized to integrate a PoP photosensitizer for the advancement of improved peptide-based cancer vaccines [12]. The final liposome formulation included 53.5 mol.% 1,2-distearoly-sn-glycero-3-phosphocholine (DSPC), 40 mol.% cholesterol, 5 mol.% 1,2-distearoyl-sn-glycero-3-phosphoethanolamine-N-[methoxy(polyethylene glycol)-2000 (DSPE-PEG2k), and 1.5 mol.% PoP. The liposome formulation process involves dissolving PoP, DSPE-PEG2k, cholesterol, and DSPC in ethanol at 60 °C, then directly injecting the hot lipids into a stirring 250 mM ammonium sulfate solution, also at 60 °C. The 20 mg/mL crude liposome suspension was then processed through a high-pressure nitrogen extruder using polycarbonate membranes (pore sizes of 0.2, 0.1, and 0.08 µm) for 10 sequential passes. To eliminate free ethanol and ammonium sulfate, the suspension underwent dialysis against a 10% sucrose solution containing 10 mM L-Histidine at pH 6.5, with at least 2 buffer exchanges. Doxorubicin loading was then achieved by incubating the liposomes with free drug at 60 °C for one hour. Liposome size and polydispersity were determined via dynamic light scattering by diluting the sample 500× in PBS. The drug loading efficiency was determined by performing size exclusion chromatography. Briefly, liposomes were diluted in PBS and passed over a Sephadex G-75 (Sigma-Aldrich, St. Louis, MO, USA) column. 24 × 1 mL fractions were collected. The first 10 fractions represent liposomal fractions, which can be used to calculate the percentage of drug contained in particles. A loading efficacy exceeding 90% is preferred, and the collected fractions can be analyzed for fluorescence on a TECAN Safire microplate reader (Morgan Hill, CA, USA). For Dox, an excitation of 480 nm and an emission of 590 nm were used. PoP measurements were done using an excitation of 420 nm and an emission of 670 nm. Drug concentration measurements for the final formulation were determined via plate reader analysis as well. Liposomes were dissolved completely in ethanol prior to plate reader measurement. Finally, liposome stability was determined by incubating the particles in 50% fetal bovine serum (FBS) at 37 °C for 6 h. Dox release was assessed by measuring fluorescence at t0 with and without Triton X-100. The final measurement was taken at t6hr to determine release via the formula Release = (F_final_ − F_initial_)/(F_X-100_ − F_initial_) × 100%.

### 2.3. Spheroid Model

**Figure 2:**
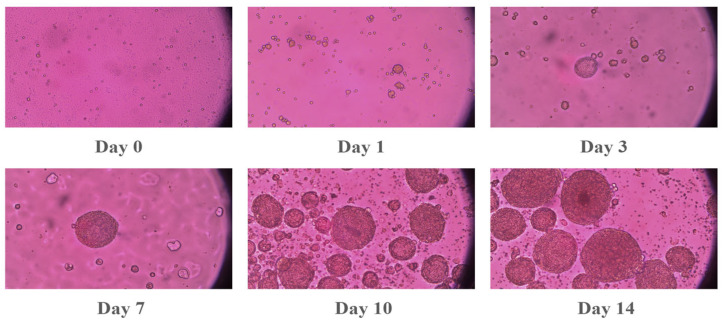
Spheroid formation of SCC2095sc cells. Spheroid formation was monitored at 8 different points (1, 3, 7, 10, and 14 days after seeding the cells). Images were obtained via an inverted microscope using a 20× objective.

**Figure 3:**
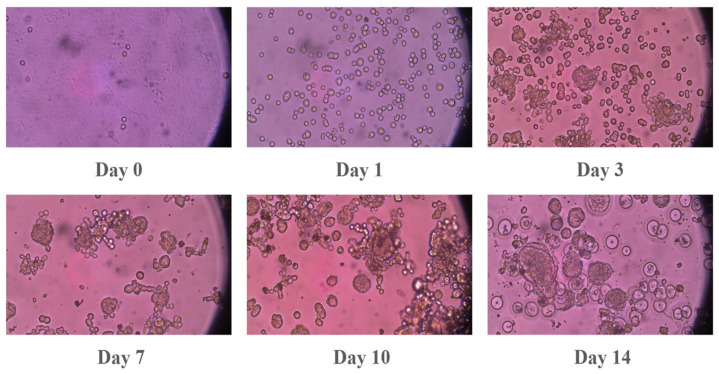
Spheroid formation of SKOV-3 cells. Spheroid formation was monitored at different time points (1, 3, 7, 10, and 14 days after seeding the cells). Images were obtained via an inverted microscope using a 20× objective.

The human epithelial ovarian adenocarcinoma cell lines (SKOV-3) were sourced from the American Type Culture Collection (ATCC, HTB-77, Manassas, VA, USA). For their maintenance, the cells were cultured in McCoy’s 5A medium, which was supplemented with 1% antibiotic–antimycotic and 10% fetal bovine serum (FBS). Human oral squamous cell carcinoma (OSCC) SCC2095sc (a gift from Professor S. Mallery, Ohio State University, Columbus, OH, USA) was cultured in Advanced DMEM-Serum Reduced Medium supplemented with 1% L-Glutamine, 5% heat-inactivated FBS, and 1% antibiotic–antimycotic. Media and supplements were purchased from Gibco (Grand Island, NY, USA). Cells were cultured at 37 °C with 5% CO_2_ in a humidified incubator. Upon reaching 80–90% confluence, cells were harvested using 0.25% trypsin-EDTA and passaged at a 1:3 ratio. To facilitate three-dimensional (3D) spheroid formation, 12-well plates were coated with Poly (2-hydroxyethyl methacrylate) (Poly-HEMA; Sigma-Aldrich, St. Louis, MO, USA) to create non-adherent surfaces. A 1.2% (*w*/*v*) Poly-HEMA working solution in 95% ethanol was applied to each well (240 µL/well) and allowed to dry for 24–48 h at room temperature. Prior to seeding, plates were sterilized via UV exposure for 30 min and rinsed twice with PBS and once with Hank’s balanced salt solution (HBSS). Spheroids were generated by seeding 10,000 viable cells per well into the Poly-HEMA-coated plates. The final volume in each well was adjusted to 1 mL with complete growth media. Spheroid morphology, growth, and integrity were monitored longitudinally (from Day 0 to Day 15) using an inverted microscope shown in Figure 2 and Figure 3. Qualitative assessments focused on spheroid shape, compactness, and the presence of cellular debris.

Chemophototherapy was performed using a porphyrin-phospholipid liposomal formulation of doxorubicin (LC-Dox-PoP) described in Section 2.4. Experiments were initiated once spheroids reached maturity, typically 14–16 days post-seeding for SKOV-3 and 10–12 days for SCC2095sc. Spheroids were treated with LC-Dox-PoP at final concentrations of 1, 3, 6, and 9 μg/mL. Control groups received complete growth medium without the drug and “blank” well plates with drug concentrations, but no cell presence was used for fluorescence signal calculation (see Section 2.4). To ensure uniform drug distribution, a designated volume of medium was replaced or supplemented with the LC-Dox-PoP working solution to reach the target concentrations. Following drug administration, plates were protected from light and incubated for 24 h. Fluorescence imaging was performed prior to and immediately after light-triggered release of LC-Dox-PoP liposomes via exposure to a 650 nm laser of 350 $mW/{cm}^{2}$ fluence for 1 min per well. After mesoscopic imaging, well plates were rinsed three times with PBS and filled with phenol-red-free media prior to well plate imaging at Ex = 480 nm, Em = 590 nm (TECAN Infinite M Plex, Morgan Hill, CA, USA).

### 2.4. Fluorescent Imaging and Image Processing

Prior to imaging the spheroid clusters, calibration images of dark signal, drug-filled wells without cells, and water-filled wells are taken at all filters to account for sensor noise, extracellular drug fluorescence, and leakages, respectively. For each experimental well, the doxorubicin and porphyrin signals were captured by illuminating the samples at their respective excitation peaks using the appropriate fluorescent and ND filters. Following this initial characterization, activation light was applied to trigger drug release, after which the fluorescent signals were re-imaged to quantify the change in localized drug concentration.

To correct for excitation light leakage into the fluorescence detection channel, an excitation-only reference image was acquired immediately prior to each fluorescence image under identical excitation conditions using a neutral-density (ND) filter with the same optical density (OD) value as the respective porphyrin and doxorubicin fluorescent filters. After dark subtraction, the ND image is assumed to be a linearly scaled version of leakages in the image taken with the fluorescent filters with the same OD blocking filters. To derive this scaling factor, calibration images without fluorophores (water-filled wells of identical volume to cell wells) were taken prior to fluorescence imaging at all filter configurations, where the true fluorescence signal is 0. We then perform an Ordinary Least Squares regression over these images to determine our scaling factor *α*:

(1) $$\alpha =\;\frac{\sum_{\left(x,y\right)}F\left(x,y\right)\cdot ND(x,y)}{\sum_{\left(x,y\right)}{\left(ND\left(x,y\right)\right)}^{2}}$$

where (*x,y*) is the set of pixel coordinates for the *ND* and fluorescent (*F*) images. To prevent overcorrection, a spatially weighted mask (M) is derived from the normalized signal from the ND filter. Then, the weighted and scaled leakage estimate is subtracted from the processed fluorescence image:

(2) $$F_{corr}\left(x,y\right)=F\left(x,y\right)-[\alpha \;\cdot ND\left(x,y\right)\cdot M\left(x,y\right)]$$

This method corrects for leakage at the cell level, which would be standard across the well plate, and leakage due to reflections caused by the plastic well plate material that could confound the signal. This approach preserves linearity, accounts for spatially localized leakage, and minimizes subtraction of the true fluorescence signal.

**Figure 4:**
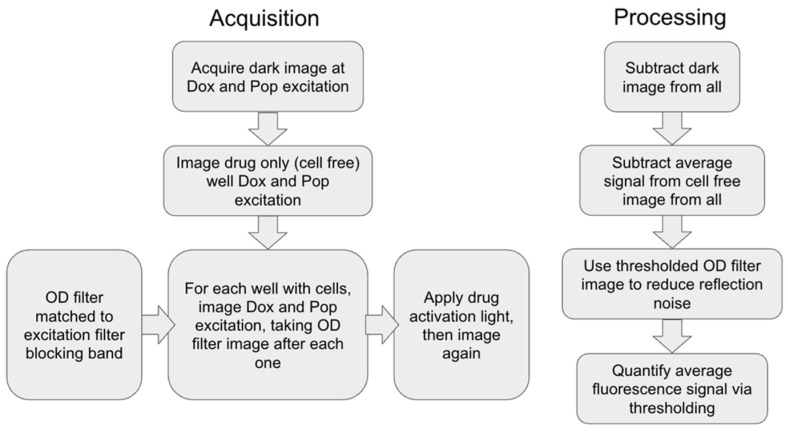
Flowchart of fluorescent imaging and post-processing.

Final quantification of the drug signal was achieved through segmentation, defined by the selection of pixels with intensity values exceeding a threshold that corresponded to the background fluorescence of the culture media. This segmentation process ensured that the average signal intensity reflected only the drug accumulated within the cellular structures, providing a representation of the uptake and release kinetics within the spheroid clusters specifically. Image acquisition and post processing is summarized in Figure 4.

Image processing parameters were determined empirically from calibration datasets acquired prior to each imaging session. The OLS scaling factor α derived from water-filled calibration wells (*n* = 4 wells per session) imaged at each filter configuration; across sessions, α converged to a mean of 0.91 ± 0.03 (R^2^ = 0.976 ± 0.008), indicating that approximately 91% of the leakage signal present in the fluorescence channel was linearly accounted for by the co-acquired ND reference image. Residual variance in α across spatial positions was captured by the spatially weighted mask, which effectively suppressed over-correction in low-intensity peripheral regions of the well. Segmentation thresholds were defined as the mean background fluorescence intensity of cell-free culture media plus three standard deviations, measured from regions of interest outside the spheroid cluster boundaries in each well. For SCC spheroids, this threshold was set at 480 ± 45 A.U., corresponding to roughly 3.2% of the maximum observed signal. For SKOV-3 spheroids, the threshold was set at 1150 ± 90 A.U. (∼2.9% of maximum signal), reflecting the higher background fluorescence observed in SKOV-3 wells under our imaging conditions in the 2D cell model. These thresholds were held constant across pre- and post-activation imaging timepoints within each experiment to ensure that any change in segmented mean intensity reflected genuine drug redistribution rather than a shift in the segmentation boundary.

## 3. Results

### 3.1. Fluorescence Calibration in 2D Cell Culture

**Figure 5:**
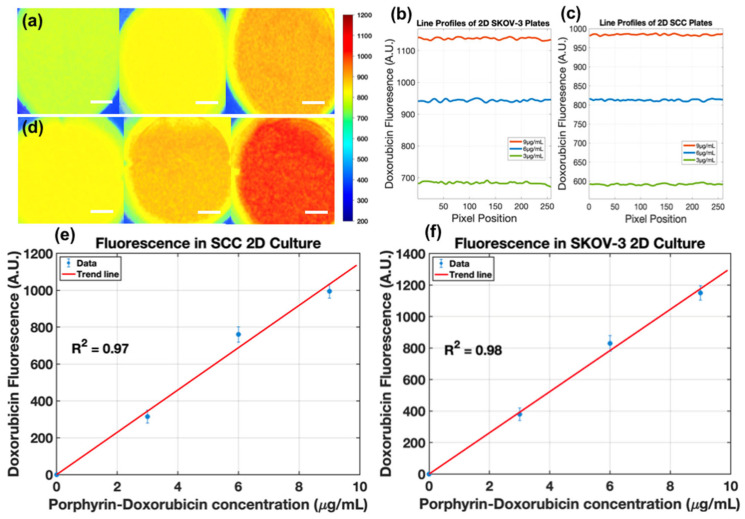
Fluorescence imaging and analysis of doxorubicin–porphyrin liposome uptake. (**a**) SCC2095sc and (**d**) SKOV-3 cells in 2D culture treated with 3, 6, and 9 μg/mL of liposomes following 1 min of light activation (scale bar = 700 μm). (**b**,**c**) Corresponding fluorescence line profiles obtained from the center of the well plates for SCC2095sc and SKOV-3 cells, respectively. (**e**,**f**) Linear regression analysis showing a strong correlation between concentration and fluorescence for both SCC2095sc (R^2^ = 0.97) and SKOV-3 (R^2^ = 0.98). (Image processing details see Appendix A and Supplementary Material).

Fluorescence imaging in 2D culture demonstrated concentration-dependent accumulation of light-activated LC-Dox-PoP within both cell lines. SKOV-3 cells exhibited moderately higher signal intensity than SCC2095sc across matched drug concentrations (Figure 5a–d). As shown in Figure 5e,f, both models exhibited a linear, dose-dependent relationship between administered LC-Dox-PoP concentration and post-activation doxorubicin fluorescence. Signal distribution within wells showed modest heterogeneity (standard deviation < 0.15 across wells), consistent with predominantly intracellular localization rather than uniform surface adsorption.

### 3.2. Doxorubicin and Porphyrin Signal in Spheroid Clusters

#### 3.2.1. SCC2095sc Spheroid Clusters

**Figure 6:**
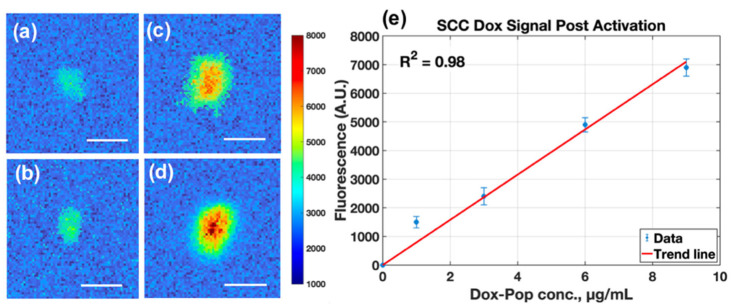
Mesoscopic fluorescent images of post-activated doxorubicin signal in SCC2095sc spheroid clusters. (**a**–**d**) Shows fluorescent image of spheroid cluster with cultured drug concentrations of 1, 3, 6, and 9 μg/mL (scale bar = 300 μm). (**e**) Displaying the increase in fluorescence signal from spheroid clusters within each well demonstrating a linear trend (R^2^ = 0.98) with respect to concentration. (Image processing details see Appendix A and Supplementary Material).

In SCC2095sc spheroid clusters, post-activation doxorubicin fluorescence increased linearly with administered LC-Dox-PoP concentration (1–9 μg/mL), indicating scalable uptake and triggered release without saturation over this range (Figure 6e, R^2^ = 0.98). Representative mesoscopic images (Figure 6a–d) show localized drug-associated fluorescence within spheroid aggregates, with heterogeneous intracluster distribution that is expected in 3D transport-limited geometries. SCC2095sc is a human oral squamous cell carcinoma line, supporting extension of this release-monitoring workflow to head and neck/oral cancer models.

**Figure 7:**
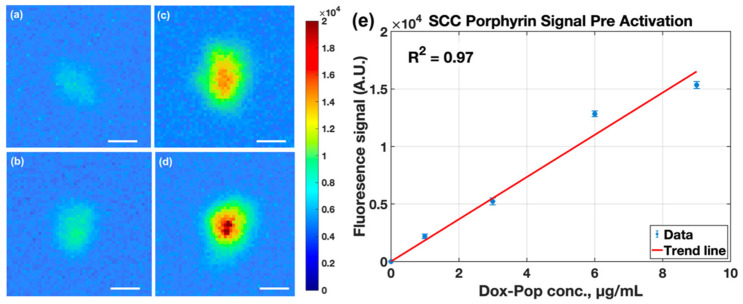
Mesoscopic fluorescence images of pre-activated porphyrin (PoP) signal in SCC2095sc spheroid clusters, with administered LC-Dox-PoP concentrations of 1, 3, 6, and 9 μg/mL in (**a**–**d**), displaying a PoP signal maps (scale bar = 300 μm). (**e**) Mean thresholded porphyrin fluorescence increased linearly with administered concentration (R^2^ = 0.97).

Pre-activation porphyrin fluorescence exhibited a linear dose–response relationship, confirming consistent uptake of the liposomal carrier across the tested concentrations (Figure 7e). The porphyrin channel provided higher raw fluorescence counts and clearer localization of spheroid aggregates than the doxorubicin channel under our acquisition settings (compare Figure 6 and Figure 7), consistent with the far-red emission band of PoP (~720 nm) and prior reports of strong porphyrin fluorescence in PoP formulations [9,10,11,12,13,14,51]. Because the two channels use different excitation/emission bands and acquisition parameters, cross-channel comparisons should be interpreted qualitatively. In this framework, PoP fluorescence primarily supports carrier localization/treatment planning, while doxorubicin fluorescence reports triggered release.

#### 3.2.2. Doxorubicin and Porphyrin Signals in SKOV-3 Spheroid Clusters

**Figure 8:**
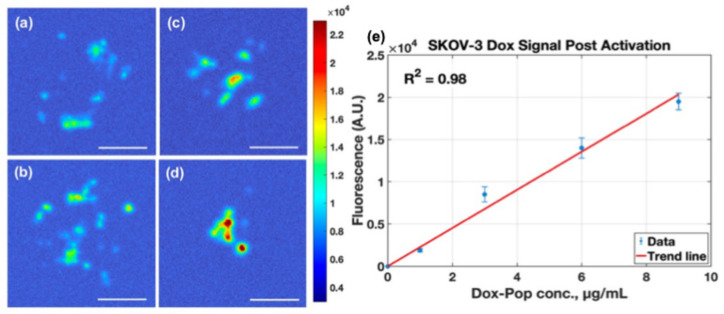
Mesoscopic fluorescent images of post-activated doxorubicin signal in SKOV-3 spheroid clusters. (**a**–**d**) Shows fluorescent image of spheroid cluster with cultured drug concentrations of 1, 3, 6, and 9 μg/mL (scale bar = 300 μm). (**e**) Displaying the increase in fluorescence signal from spheroid clusters within each well demonstrating a linear trend (R^2^ = 0.98) with respect to concentration.

In contrast to the compact SCC2095sc spheroids, SKOV-3 clusters exhibited a more dispersed morphology. Despite this structural heterogeneity, post-activation doxorubicin fluorescence increased progressively with administered LC-Dox-PoP concentration (Figure 8e), and digital zoom images highlight non-uniform intracluster drug distribution (Figure 8a–d). For visualization of small, spatially distributed clusters, the zoomed panels are displayed with locally scaled color maps. Quantitative comparisons were performed on the underlying leakage-corrected fluorescence images using consistent thresholding to preserve linearity across concentrations.

**Figure 9:**
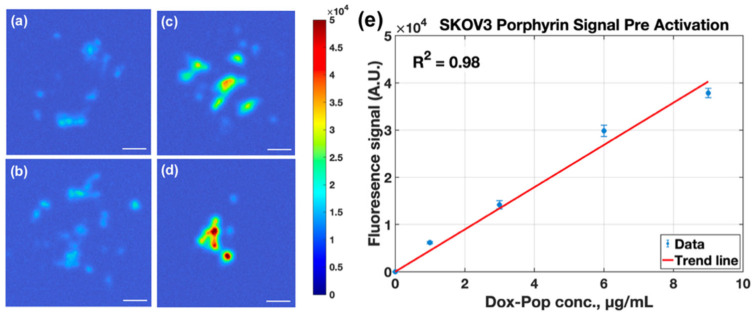
Mesoscopic fluorescence images of pre-activated porphyrin (PoP) signal in SKOV-3 spheroid clusters, with administered LC-Dox-PoP concentrations of 1, 3, 6, and 9 μg/mL in (**a**–**d**), displaying a PoP signal maps (scale bar = 300 μm). (**e**) Mean thresholded porphyrin fluorescence increased linearly with administered concentration (R^2^ = 0.98).

In SKOV-3 spheroid clusters, pre-activation porphyrin fluorescence also scaled linearly with administered LC-Dox-PoP concentration (Figure 9e). Under the acquisition settings used here, porphyrin fluorescence exhibited a higher dynamic range than doxorubicin fluorescence (compare Figure 8 and Figure 9), which is consistent with the strong far-red PoP emission and reduced background at longer wavelengths. SKOV-3 clusters also demonstrated higher mean fluorescence than SCC2095sc in both channels, which likely reflects differences in spheroid morphology, uptake, and/or cell density (see Section 4).

### 3.3. Validation of Fluorescence Signal

**Figure 10:**
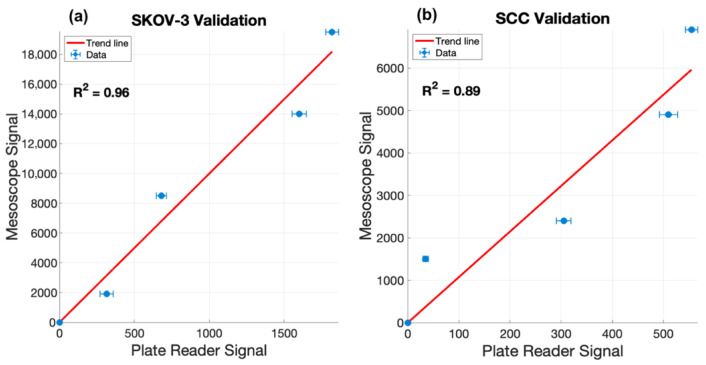
Validation of laparoscopic doxorubicin fluorescence measurements against a standard well-plate reader for SKOV-3 (**a**) and SCC2095sc (**b**) spheroid clusters. Linear regression yielded R^2^ = 0.96 (SKOV-3) and R^2^ = 0.89 (SCC2095sc) across the tested LC-Dox-PoP concentrations.

To validate the accuracy of the laparoscopic fluorescence workflow, mean doxorubicin fluorescence from each concentration group was compared against standard plate reader measurements. In Figure 10, a linear regression demonstrated strong agreement R^2^ = 0.96 (SKOV-3) and R^2^ = 0.89 (SCC2095sc) between platforms for both cell models, supporting the use of the laparoscope-derived signal as a quantitative readout of drug-associated fluorescence in the spheroid assay.

**Figure 11:**
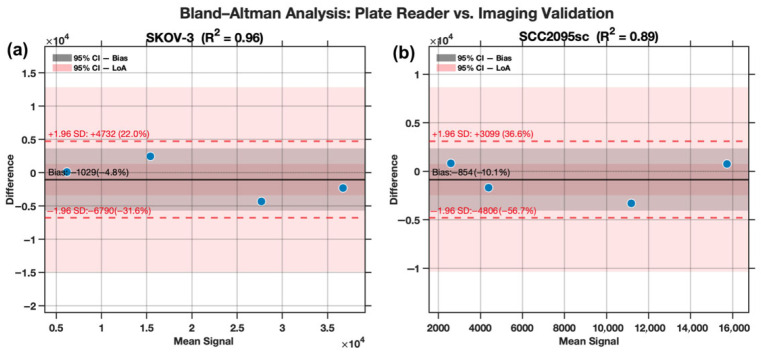
Bland–Altman analysis of plate reader versus laparoscopic mesoscope signal quantification in SKOV-3 and SCC2095sc tumor spheroid models with the paired measurements from the laparoscope and well plate reader plotted in blue. Mean signal values are plotted against inter-platform differences for (**a**) SKOV-3 (R^2^ = 0.96, *n* = 4) and (**b**) SCC2095sc (R^2^ = 0.89, *n* = 4). Solid black lines indicate the mean bias; dashed red lines denote the upper and lower 95% limits of agreement (±1.96 SD). Shaded regions represent 95% confidence intervals around the bias (gray) and limits of agreement (red). A consistent negative bias in both cell lines indicates systematic underestimation of fluorescence signal by the plate reader relative to mesoscopic imaging, with greater inter-platform discordance observed in the SCC2095sc spheroid model.

Bland–Altman analysis was used to characterize systematic bias and inter-platform variability between bulk fluorescence plate reader measurements and spatially resolved laparoscopic mesoscope imaging across two morphologically distinct spheroid models shown in Figure 11. In SKOV-3 spheroids, which adopt a loosely aggregated architecture with comparatively unobstructed fluorophore accessibility, inter-platform correlation was strong (R^2^ = 0.96), and the mean bias was modest at −1029 signal units (−4.8% of the mean signal), suggesting that the plate reader captures bulk fluorescence with reasonable fidelity when light scattering and inner-filter effects are limited by open spheroid structure. In contrast, SCC2095sc spheroids—which form tightly packed, optically dense aggregates—exhibited a notably weaker inter-platform correlation (R^2^ = 0.89) and a proportionally larger negative bias of −853.5 units (−10.1%), despite the smaller absolute magnitude. This pattern is consistent with the known optical limitations of plate-based fluorimetry when applied to compact, high-density spheroids: the bulk-averaging geometry of the plate reader is more susceptible to self-quenching, inner-filter attenuation, and fluorescence reabsorption within the spheroid core, all of which are exacerbated by tight cellular packing. The laparoscopic mesoscope, by contrast, resolves signal spatially across the spheroid surface and periphery, circumventing core attenuation and yielding systematically higher fluorescence estimates. The wider limits of agreement in SKOV-3 (total LoA width: 11,521.9 units) relative to SCC2095sc (7904.9 units) likely reflect greater spheroid-to-spheroid size variability in the loosely aggregated SKOV-3 model, which amplifies scaling differences between platforms at higher signal magnitudes.

### 3.4. Porphyrin Signal in Spheroid Clusters

**Figure 12:**
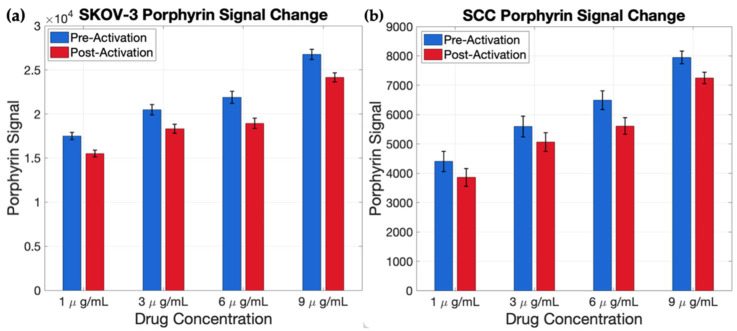
Averaged porphyrin signal value from SKOV-3 (**a**) and SCC2095sc (**b**) spheroids before and after NIR light-induced release, showing a slight decrease after light exposure.

Across both SKOV-3 and SCC2095sc spheroid clusters, mean porphyrin fluorescence decreased modestly after 660 nm activation light exposure at all tested concentrations (Figure 12), consistent with PoP photobleaching during CPT illumination. This behavior provides complementary feedback such that pre-activation PoP fluorescence can be used to localize nanocarrier accumulation for treatment planning, while a post-illumination decrease in PoP signal can serve as an indicator of delivered light dose/activation, alongside the doxorubicin release readout.

The porphyrin signal from the LC-Dox-PoP liposomes was also measured before and after light-triggered release. There was a consistent decrease in porphyrin fluorescence after drug release, suggesting a degree of photobleaching occurring during the light-delivery process. Despite this slight attenuation, the porphyrin signal remained a robust indicator of the initial liposomal carrier distribution throughout the 3D spheroid architecture, with a spatial distribution that closely mirrored the doxorubicin localization within both the SCC and SKOV-3 clusters.

### 3.5. Microscopic Spheroid Clusters

**Figure 13:**
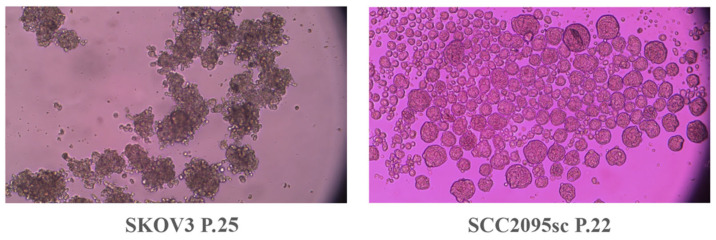
Comparison of spheroid formation in SCC2095sc and SKOV-3 cells 10 days after seeding, demonstrating distinct morphological characteristics. Images were acquired using a 10× objective.

Figure 13 compares spheroid morphology between SKOV-3 and SCC2095sc cell lines 10 days after seeding. SKOV-3 cultures form fewer uniform aggregates with smaller clusters and dispersed cellular material, whereas SCC2095sc forms densely packed, rounded spheroids of varying sizes. These morphological differences likely contribute to the observed differences in fluorescence intensity and spatial distribution between models by altering transport barriers and effective cell density within the imaged clusters.

### 3.6. Characterization of Doxorubicin-Loaded PoP Liposomes

The liposomes for this study were prepared and characterized as described in Section 2.2. Following drug loading and sterile filtration, sample concentration measurements were determined. The liposomes were found to have a PoP concentration of 0.37 mg/mL and a Dox concentration of 2.05 mg/mL. Particle size was found to be 86.45 ± 1.01 nm, with a polydispersity index of 0.082 ± 0.046. Drug encapsulation efficiency was determined using size exclusion chromatography, which revealed 92.9% Dox loading for these particles. Finally, particle stability was determined by diluting liposomes in 50% fetal bovine serum and assessing drug leakage after 6 h of incubation at 37 °C. The liposomes showed only 2.9% drug release over the course of the experiment, demonstrating high stability.

## 4. Discussion

SCC2095sc cells consistently formed dense, uniform, and spherical aggregates characterized by strong cell–cell junctions and a smooth boundary. In contrast, SKOV-3 spheroids exhibited a dispersed morphology with greater variability in size and shape, likely due to reduced expression of adhesion molecules and a more fragmented extracellular matrix. These morphological differences [52] directly influenced the varying fluorescence signals observed between the two cell types. The compact, high-density packing of SCC2095sc spheroids created physical barriers and high interstitial pressure [53] that limited drug diffusion and reduced the final signal relative to SKOV-3 spheroids. Conversely, the porous and irregular architecture of SKOV-3 clusters provided a higher effective surface area and reduced transport resistance, allowing for improved penetration of the liposomes and a higher recorded signal. Furthermore, the similarity in drug uptake profiles between SCC2095sc and SKOV-3 cells in two-dimensional culture suggests that the differences observed in the three-dimensional models are structural rather than purely biochemical.

In this model, the higher fluorescence signal observed in the spheroid center versus the periphery and 2D monolayers is likely an artifact of optical integration. Because widefield mesoscopic imaging projects 3D volumes into 2D images, the signal is integrated along the entire optical path length [54,55,56]. Thus, the thicker center of a spheroid and its stacked cellular layers yield a much higher cumulative intensity than the thinner edges or a single cell layer. Additionally, the central signal concentration likely reflects the 24 h incubation period, which provides sufficient diffusion time for the necrotic core to act as a metabolic “sink” [57,58], this model lacks the vasculature and immune components that define in vivo transport. This represents a key distinction from in vivo tumors, where high interstitial pressure, dense matrices, and limited blood flow often prevent doxorubicin from reaching the tumor center, highlighting a limitation in how spheroid models replicate complex physiological barriers. Future validation using spatial drug-mapping approaches, such as MALDI-MS, would be valuable to correlate these optical signals with absolute molecular distribution, though such analysis falls beyond the scope of this instrumentation study.

The linear increase in signal as a function of administered LC-Dox-PoP concentration indicates that the uptake and delivery mechanism remains within a linear kinetic range in these assays. Across a 1–9 μg/mL range, the system did not reach an apparent saturation point for carrier accumulation and/or drug-associated fluorescence [59], suggesting a predictable and scalable dose–response relationship in three-dimensional tumor models.

The experimental results demonstrated a clear correlation between the fluorescence signal and the concentration of the delivered therapeutic. In both the 2D monolayer and the 3D spheroid models [60], a proportional relationship was observed in the signals recorded from the samples, which should be interpreted as a controlled in vitro proof-of-concept over the tested range. This study intentionally isolates the filtered fluorescence workflow in lower-scattering in vitro systems before introducing optical-property correction in more complex environments. A logical next step for clinical translation involves integrating the laparoscopic platform’s SFDI capability with tissue-mimicking phantoms and heterogeneous in vivo models. This laparoscopic workflow first localizes PoP-associated carrier accumulation, delivers controlled activation light, and finally monitors Dox fluorescence as a readout of local release. Full clinical translation must address motion management, optical-property correction in heterogeneous tissues, and sterile packaging validated under relevant illumination geometries. Further studies across a broader range of concentrations are required to establish a robust calibration curve [23]. The use of light-triggered doxorubicin in conjunction with a filtered laparoscopic system provides a robust framework for fluorescence-guided surgery. Additionally, the spheroid model here provides a more robust estimate of in vivo drug kinetics due to the more realistic properties of the tumor microenvironment [43,44,45]. By successfully isolating the signals of Dox in light-triggered liposomes and subtracting background artifacts via ND filtering, we have shown that it is possible to visualize simulated micromet cell clusters in an in vitro model.

Importantly, the strong linearity of the fluorescence dose–response across both 2D and 3D models (R^2^ ≥ 0.97) and the cross-platform agreement with plate reader measurements (R^2^ = 0.89–0.96) indicate that the laparoscopic workflow can provide a quantitative readout of nanocarrier delivery and light-triggered release. In the context of chemophototherapy, such fluorescence-based feedback could enable lesion-specific treatment planning (carrier localization via PoP) and real-time monitoring of on-target drug activation (Dox release), supporting optimization of light dosimetry while reducing off-target exposure. Moreover, the higher signal levels observed in the porphyrin channel relative to doxorubicin under our imaging conditions suggest that PoP fluorescence may be more robust for intraoperative localization and treatment planning, whereas doxorubicin fluorescence remains valuable as a release-specific reporter but may be more susceptible to attenuation and background at shorter wavelengths. This concept is consistent with prior demonstrations of imaging-guided PDT using porphyrin-based nanoliposomal platforms, where fluorescence readouts were used to localize therapeutic accumulation and guide light delivery [36]. Moreover, time-gated fluorescence tomography has been used to image PDT photosensitizer distributions in vivo, providing a route toward depth-sensitive intensity/lifetime quantification in future extensions of this workflow [27].

In this study, the PoP signal decrease serves as a quantitative complementary indicator of light delivery across the tested 1–9 µg/mL concentration range. While this provides a proportional measure of activation, it is not yet a standalone dosimetry metric. However, moving to in vivo applications will require optical-property correction factors (to account for tissue scattering and absorption) and oxygen-sensitive readouts to calculate the absolute photochemical yield.

Beyond peritoneal disease, the inclusion of an oral squamous cell carcinoma model (SCC2095sc) highlights the potential applicability of this quantitative fluorescence workflow to head and neck/oral cancer. In head and neck surgery, local recurrence is often driven by microscopic residual disease; a locally activated, fluorescence-monitored chemophototherapy strategy could complement postoperative adjuvant PDT or chemotherapy by confining activation to the surgical bed while limiting off-target exposure. Future studies will translate these in vitro findings to orthotopic head and neck models and evaluate treatment-response monitoring under clinically relevant illumination geometries. Consistent with this rationale, Mallidi and colleagues have evaluated intraoral PDT light-delivery systems for oral lesions and have reported head and neck/oral cancer 3D spheroid models for quantitative evaluation of photoactive agents and image-guided phototherapies [28,46,47,48]. In addition, studies on HPPH-based porphyrin analogs have shown that peripheral substitutions (e.g., charge/lipophilicity) can substantially influence cellular uptake/retention and PDT efficacy, motivating quantitative fluorescence readouts for selecting and optimizing photoactive agents for specific tumor contexts [61]. Additionally, photoactivated HPPH-liposomes have demonstrated tumor-selective uptake and phototherapy-induced tumor control in chemoradioresistant head and neck cancer patient-derived xenograft models, supporting translation of porphyrin-liposomal photoactivated delivery strategies [62]. Clinical studies of HPPH-mediated photodynamic therapy in oral cavity/head and neck cancer further support the feasibility of porphyrin-based, locally activated adjuvant approaches [63,64,65,66,67,68,69,70,71,72].

## 5. Conclusions

This study demonstrates a filtered laparoscopic fluorescence imaging workflow for quantitative monitoring of light-triggered LC-Dox-PoP delivery in 2D cultures and 3D spheroid clusters. Dox fluorescence scaled linearly with administered concentration (R^2^ = 0.97–0.98 in 2D and R^2^ = 0.98 in spheroid clusters) and agreed with standard plate reader measurements (R^2^ = 0.89–0.96). Complementary PoP fluorescence supported localization of nanocarrier distribution and remained detectable after activation despite modest photobleaching. Together, these results support dual-fluorescence PoP liposomes as a theranostic platform and establish quantitative imaging readouts that could guide lesion-specific light dosimetry and monitor treatment response during intraoperative chemophototherapy for peritoneal micrometastases. Future work will evaluate in vivo performance and incorporate optical-property correction strategies to improve quantitative robustness in heterogeneous tissue. Demonstrating performance in both SKOV-3 ovarian and SCC2095sc oral squamous cell carcinoma spheroids supports broader translation of fluorescence-guided chemophototherapy monitoring to additional surgical adjuvant settings, including head and neck/oral cancer [62,66,68].

## Figures and Tables

**Table 1:** Comparison of the FOV, resolution, and depth of field of the standard laparoscope vs. the proposed.

Laparoscope	Field of View	Resolution	Depth of Field
Standard laparoscope [50]	25–40 cm^2^	2–5 lp/mm	5 cm
Proposed	44 cm^2^ (2×: 26 and 3×: 10.2 cm^2^)	1.96 lp/mm (2×: 2.52 and 3×: 7.14 lp/mm)	2–7 cm

## Data Availability

Data presented in this study is contained within the article. Further inquiries can be directed to the corresponding author.

## Supplementary Materials

The following supporting information can be downloaded at: https://www.mdpi.com/article/10.3390/pharmaceutics18040495/s1, The original images corresponding to Figure 5d (SKOV, 9 μg/mL), Figure 5a (SCC, 9 μg/mL), Figure 6a,d.

## Appendix A

### Appendix A.1. Image Processing Details Corresponding to Figure 5d (SKOV 9 µg/mL), Figure 5a (SCC 9 μg/mL)

(1) Images were originally acquired at 14-bit intensity depth using an Andor Luca EMCCD camera and subsequently upsampled to 16-bit prior to processing. This bit-depth conversion is performed by left-shifting the 14-bit values by two bits to fill the 16-bit container, which pads the two least significant bits of every pixel with zeros. As a result, the effective intensity precision remains 14-bit despite the wider bit depth, and the populated intensity values are distributed across the 16-bit range in a structured, non-contiguous pattern, meaning many intensity levels in the 16-bit space are never occupied. This intrinsic quantization means that pixels with subtly different original intensities may already map to identical 16-bit values before any further processing occurs, seeding a tendency toward repeating intensity regions.
(2) To further improve the visibility of low-intensity cell signal in the 2D well culture, a spatial upscaling step was then applied to expand the dynamic range and increase the spatial footprint of dim features before contrast stretching and intensity adjustment were performed. When nearest-neighbor interpolation (the default resampling method in many image analysis platforms) is used during this upscaling, each output pixel is assigned the exact value of its nearest source pixel with no blending between neighbors. This means that discrete cell signal features, such as localized fluorescence points or cell body signal, are directly replicated across multiple contiguous output pixels, producing regions of cell signal that appear strikingly similar to one another.
(3) After spatial upscaling, pixels falling below a defined intensity threshold were replaced with values sampled from the pool of pixels residing above that threshold (as described in figure).
(4) This step was implemented to normalize signal heterogeneity across the well and reduce the contribution of dim, sub-threshold pixels to downstream intensity analysis. However, because the replacement values are drawn by sampling from a finite pool of above-threshold pixel intensities and redistributed across a potentially large number of sub-threshold pixels, the same source intensity values are necessarily reused at multiple spatial locations throughout the image. This introduces regions of identical or near-identical pixel intensity across the image that are not present in the original acquisition, and which can manifest as visually repeating patches of signal, particularly in areas where the density of above-threshold pixels is low and the same sampled values are applied repeatedly.
(5) Lastly, subsequent conversion of the processed image to PNG format reduces the 16-bit intensity values to an 8-bit integer scale, which can cause subtly different pixel intensities that survived the prior steps to collapse to identical discrete values, further reinforcing the visual similarity between already-replicated signal regions. The raw pre-processing images contain entirely unique pixel values at every acquisition coordinate.

### Appendix A.2. Image Processing Details Corresponding to Figure 6a,d

(1) Both images were acquired at 14-bit intensity depth using an Andor Luca EMCCD camera and upsampled to 16-bit prior to processing, a conversion achieved by left-shifting the 14-bit values by two bits and padding the least significant bits with zeros. This means that despite occupying a 16-bit container, the intensity values remain constrained to a 14-bit precision and are distributed non-contiguously across the 16-bit range, such that many intensity levels are structurally absent. In low-signal background regions where pixel intensities are already clustered near the noise floor, this quantization causes many background pixels across both images to resolve to the same small set of 16-bit values, introducing an initial layer of intensity repetition before any spatial processing occurs.
(2) A spatial upscaling step was then applied to both images using nearest-neighbor interpolation to increase the effective resolution of dim spheroid edge features prior to contrast enhancement. This replicates each source pixel’s value (including its quantized background intensity) directly into multiple contiguous output pixels without blending, causing the background speckle noise to be copied in a structured, block-wise fashion across both images. Because both spheroids were acquired under identical conditions (matched gain, exposure, and culture matrix) their backgrounds share a statistically similar noise profile from the outset, and the nearest-neighbor resampling amplifies this similarity into visually near-identical repeating texture.
(3) The subsequent conversion of both images to PNG format further consolidates this appearance, as the reduction to an 8-bit integer intensity scale causes the fine gradations in background speckle intensity to be quantized to identical discrete values, rendering the background regions of both figures visually indistinguishable despite originating from independent acquisitions.

The original images corresponding to Figure 5d (SKOV, 9 μg/mL), Figure 5a (SCC, 9 μg/mL), Figure 6a,d can be accessed in the Supplementary Materials.
