# Supplementary material for: Mesoscopic Fluorescence Imaging of Light-Triggered Chemotherapeutic Release in Cancer Spheroid Models
Source: Pharmaceutics. 2026 Apr 17;18(4):495. doi: 10.3390/pharmaceutics18040495 (PMC13118955; doi:10.3390/pharmaceutics18040495)

The original images corresponding to Figure 5d (SKOV, 9 μg/mL)

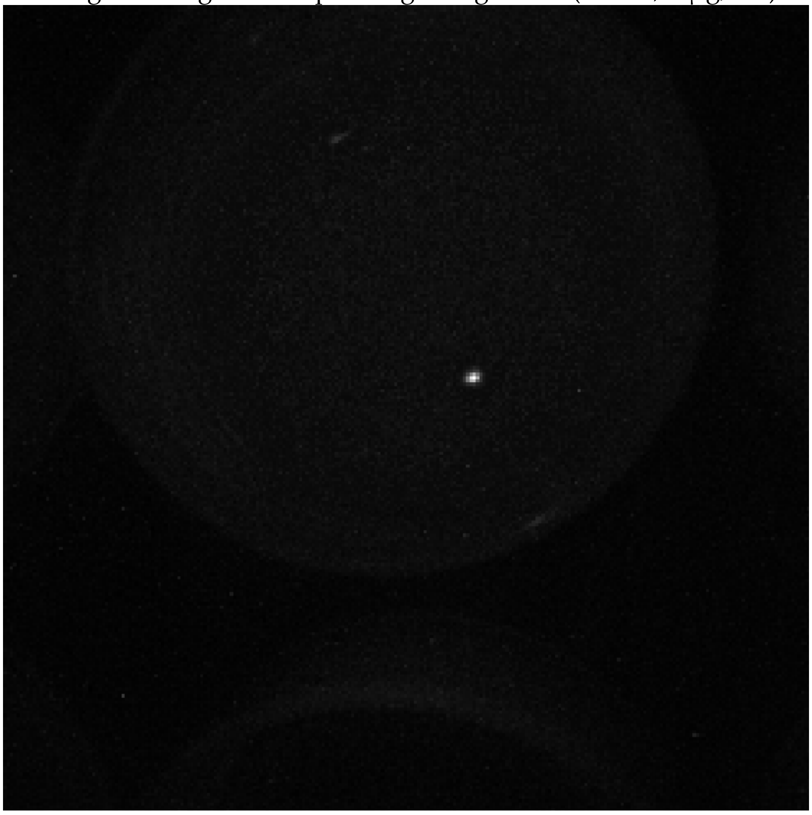

The original images corresponding to Figure 5a (SCC, 9 μg/mL)

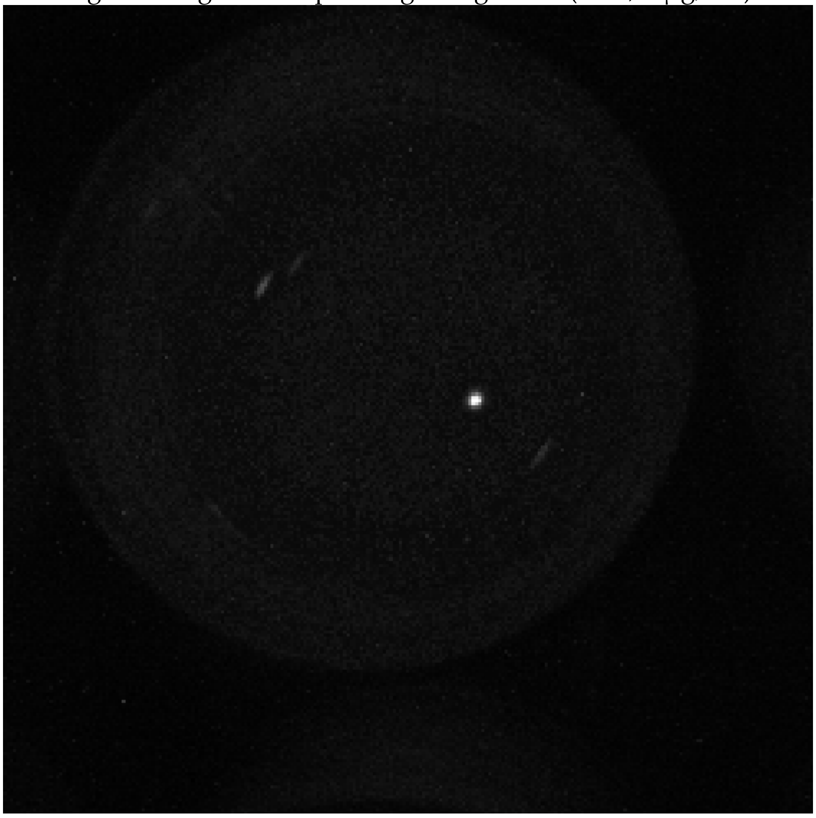

The original images corresponding to Figure 6d

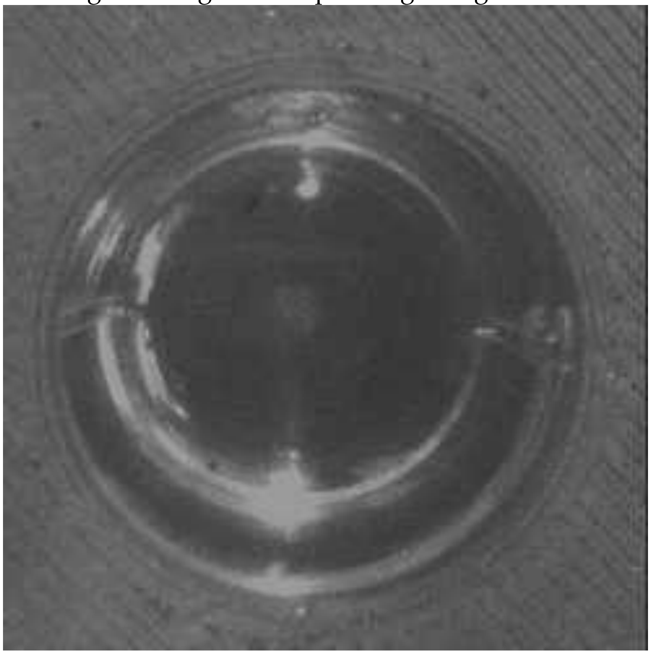

The original images corresponding to Figure 6a

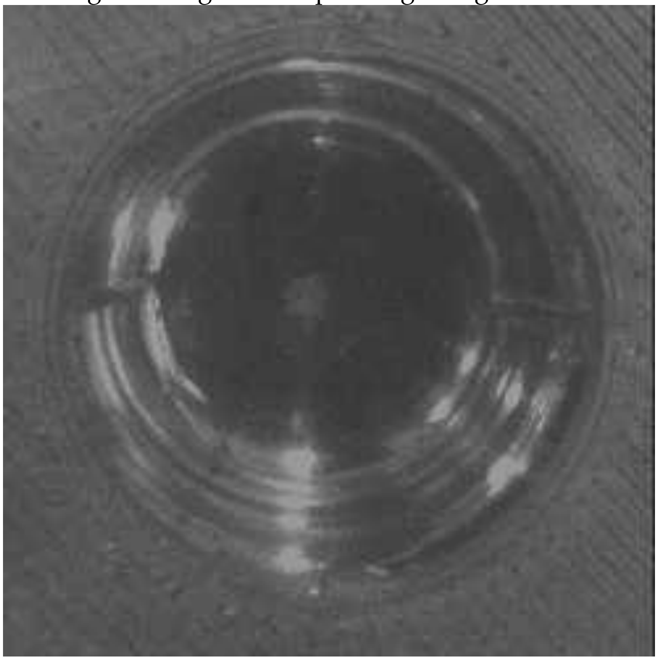

Supplement: Supplementary file 1 [file pharmaceutics-18-00495-s001.zip › pharmaceutics-4184404-supplementary.pdf]
